# Comparison of Pain Levels With Postoperative Intramuscular Administration of Single-Dose Ketoprofen Versus Diclofenac Sodium in Patients Undergoing Lower Third Molar Surgery

**DOI:** 10.7759/cureus.47499

**Published:** 2023-10-23

**Authors:** Manishaa V, Senthil Murugan P, Saravanan Lakshmanan, Murugesan Krishnan, Santhosh P Kumar, Sibashish Khuntia

**Affiliations:** 1 Oral and Maxillofacial Surgery, Saveetha Dental College and Hospitals, Saveetha Institute of Medical and Technical Sciences, Saveetha University, Chennai, IND

**Keywords:** pain levels, ketoprofen, diclofenac, third molar surgery, impaction

## Abstract

Background

Third molar surgeries are commonly performed in oral and maxillofacial surgery practice. Pain associated with this procedure is usually a frequent reason for patient apprehension and discomfort. Oral analgesics, though effective, do not provide sufficient pain relief in the immediate postoperative period.

Aim

To assess the postoperative effect on pain levels of single-dose administration of ketoprofen and diclofenac sodium as an injection in patients undergoing third molar removal surgeries.

Methods

This study was conducted among 30 patients divided into two groups (n=15). Patients in Group K received injection ketoprofen 100 mg and Group D included patients receiving injection diclofenac sodium 75 mg, both intramuscularly postoperatively. The intensity of pain was assessed at 30 minutes, two, six, and eight hours post-surgical removal of the impacted tooth using the visual analogue scale (VAS). The statistical data was analyzed using SPSS for Windows version 23.0 (IBM Corp., Armonk, NY, USA). The comparative statistical test adopted to compare pain scores between the two groups was the Independent samples t-test. A p-value of <0.05 was considered to be statistically significant.

Results

Our study results revealed that Group K patient's VAS scores after two hours, six hours, and eight hours were 3.9 ± 2.7, 4.5 ± 3.23, 2.93 ± 2.27 respectively. In Group D patient's VAS scores after two hours, six hours, and eight hours were 4.83 ± 2.82, 5.03 ± 2.9, 3.73 ± 2.91 respectively. Patients who were administered ketoprofen had lower pain levels when compared to patients who were given diclofenac but the difference was not statistically significant at any time point (p=0.172 after eight hours). Our results depicted that the pain levels uniformly reached their maximal levels six hours after the procedure and thereafter steadily declined in both groups.

Conclusion

It can be concluded from the study that both the drugs ketoprofen and diclofenac sodium analyzed in this study can be used interchangeably for the reduction of pain following lower third molar surgery.

## Introduction

Among the several procedures performed by oral and maxillofacial surgeons, the removal of mandibular third molars is the most common and routinely performed procedure. However, pain management postoperatively is of utmost importance and still an area of concern. The pain and postoperative effects worsen with an increase in the difficulty of the impactions [1]. Evidence of the systemic effects of inadequate analgesia and the consequent pain sparked interest and brought attention to the therapeutic significance of this topic [2]. Any kind of surgery will result in injury and inflammation that causes changes in the perception of any kind of stimuli. Nonsteroidal anti-inflammatory medicines (NSAIDs) have proven to be beneficial in reducing postoperative pain [3]. NSAIDs work by acting peripherally at the site of injury. NSAIDs block the mediation of the inflammatory process by inhibiting the release of prostaglandins and thromboxanes. Such metabolites do not have any systemic role and act locally [4]. NSAIDs lower the concentration of these metabolites and decrease the susceptibility of peripheral nociceptors [5].

Pain after surgical removal of impacted wisdom teeth peaks three to six hours after surgery and then subsides after 12 hours [6,7]. Hence, we prescribe postoperative analgesics to reduce the discomfort. NSAIDs decrease the frequency and intensity of symptoms like pain and swelling [8]. Compared to conventional opiate therapy, these NSAIDs produce less pain and fewer adverse effects. Common sequelae include swelling and trismus, which can last for many days. Acute pain in the temporomandibular joint region was also reported as one of the postoperative sequelae after third molar surgery, which may warrant comprehensive treatment modalities [9-11]. 

Among the NSAIDs, diclofenac is a well-researched and commonly administered drug due to its anti-inflammatory, analgesic, and antipyretic properties [12]. It has excellent analgesic efficacy and has been used widely in pain treatment following third molar surgery [13]. It functions by inhibiting the enzymes cyclooxygenase 1 and 2, which stops the synthesis of prostaglandins. Apart from diclofenac, another drug of choice for postoperative pain is ketoprofen which is commonly used in pain management following trauma and rheumatic origin [14]. Ketoprofen works by inhibiting COX, which also results in a decrease in prostaglandin synthesis.

Diclofenac and ketoprofen are efficient in providing pain relief after surgical procedures, according to placebo-controlled trials [15]. A single postoperative dose of an NSAID, such as diclofenac or ketoprofen, should minimize the inflammatory response to the trauma of surgical removal and cause a significant amount of postoperative analgesia. This study aims to assess the postoperative effect on pain levels of single-dose administration of ketoprofen and diclofenac sodium as injections in patients undergoing third molar removal surgeries. The objectives of the study were to evaluate the effectiveness of the individual drugs on pain management postoperatively and to also compare their efficacies in patients after impacted tooth removal. 

## Materials and methods

Study setting

The participants for the study were recruited from the outpatients of the Department of Oral and Maxillofacial Surgery, Saveetha Dental College and Hospital, Chennai, Tamil Nadu, India in patients presenting with a requirement for surgical removal of the impacted mandibular molar tooth. The Institutional Human Ethics committee had given approval for conducting the study (IHEC/SDC/OMFS-2104/22/186) and written consent was obtained from the participants. 

Intervention

**Figure 1:**
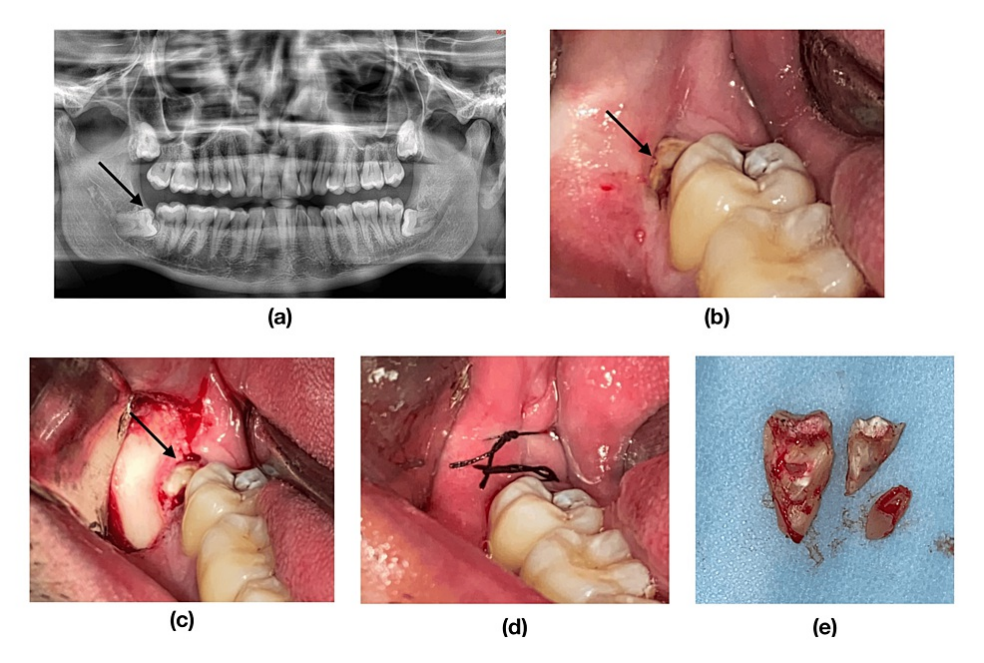
Surgical removal of impacted lower right mandibular molar tooth (a) Pre-operative orthopantomograph (black arrow indicates the impacted tooth) (b) Pre-operative intraoral photograph (c) Flap elevation (d) Closure (e) Extracted teeth

A total of 30 patients were included in the study. The sample size was determined using G power calculation on the basis of the study conducted by Velásquez et al. in 2014 [16], with a P value of 0.05 and 95 power. Each of these patients underwent surgical removal of the impacted mandibular third molar and was randomly divided into two groups. The group allocation was done based on the analgesic used postoperatively: Group K (n=15) and Group D (n=15). Group K received injection ketoprofen 100 mg intramuscular injection (IM) postoperatively and Group D received injection diclofenac sodium 75 mg IM postoperatively. A single operator performed all the surgical procedures. The patients were allocated into two groups based on sealed opaque envelopes prepared by the investigator and both the operator and the participant were unaware of the study grouping (double blinding). The mandibular anesthesia was induced with 2% lignocaine hydrochloride with 1:100,000 epinephrine using an inferior alveolar nerve block. Full thickness mucoperiosteal flap was elevated and the tooth was elevated and removed. Closure was done using 3-0 silk (Figure 1). In Group K, postoperative injection ketoprofen 100 mg IV was given as a single dose and in Group D, injection diclofenac sodium 75 mg IM was given as a single dose by the operator. 

Assessment 

All the operated patients were admitted in the day care unit and patients were discharged after assessment of postoperative pain. The pain was assessed using a Visual Analogue Scale (VAS score from 0 to 10 at 0, 0.5, two, six, and eight hours post-surgical removal of the impacted tooth, wherein a score of 0 = no pain, values between 0-3 = mild pain, values 4-7 = moderate pain, values between 8-10 = severe pain, and a score of 10 = most severe pain). Pain intensity at various time intervals was compared among the two groups. In both groups, postoperatively patients were prescribed antibiotics and analgesics for five days and 0.12% chlorhexidine gluconate mouthwash for a week. 

Inclusion criteria

Patients aged 18 years or older, presenting with Class II and position A or B, horizontal angulation (Winter's classification) impacted mandibular third molar were included. Patients who were willing for regular follow-ups were included.

Exclusion criteria

Patients presenting with systemic diseases and any infections related to the tooth of interest were also excluded.

Statistical analysis

The statistical data was analyzed using SPSS for Windows version 23.0 (IBM Corp., Armonk, NY, USA). All data were summarized as mean ± SD for continuous variables, numbers, and percentages for categorical variables. The comparative statistical test adopted to compare pain scores between the two groups was the Independent samples t-test. A p-value of <0.05 was considered to be statistically significant.

## Results

The participants of the study were segregated into two groups, with 15 in each. All the patients were similar in their socio-democratic characteristics; thus, steps have been taken to minimize the confounding factors (Table 1).

**Table 1:** Demographic data of the study participants (expressed in mean and standard deviation) Group K: Patients receiving injection ketoprofen 100 mg intramuscular (IM); Group D: Patients receiving injection diclofenac sodium 75 mg IM

DEMOGRAPHIC DATA	GROUP K	GROUP D
Age (years)	27 ± 1.3	25 ± 2.0
Gender (Male,Female)	11,19	16,14
Weight (kg)	62 ± 3.8	69 ± 1.7
Local anaesthesia (ml)	3.5 ± 0.7	3.3 ± 0.9
Duration of surgery (min)	32 ± 5.2	27 ± 3.6

All the patients included in the study had comparable pain levels preoperatively and the difference in the preoperative VAS score between groups was statistically not significant (p value > 0.05). The patients in the ketoprofen group showed a mild increase in the pain levels 30 minutes after the administration of the drug while those in the diclofenac sodium group showed comparatively lesser pain. However, these values were statistically not significant (p value = 0.192). Patients in both groups showed peak pain levels at the end of eight hours post-surgery after which the pain began to subside in both groups. Except at the start of the study, until the end of eight hours, the VAS scores were lesser in Group K when compared to Group D. Yet again, these values between the groups were statistically not significant (p value > 0.05) (Table 2). In this study, no complications or any adverse effects were reported due to these drugs.

**Table 2:** Assessment of pain at different time intervals using Visual Analogue Scale (VAS) Group K: Patients receiving injection ketoprofen 100 mg intramuscular (IM); Group D: Patients receiving injection diclofenac sodium 75 mg IM, Independent samples 't' test: p-values are statistically not significant

PAIN INTERVAL	GROUP K	GROUP D	P VALUE
Pre-operative	2.96 ± 3.07	3.01 ± 3.26	0.210
After 30 minutes	3.33 ± 3.53	2.8 ± 2.95	0.192
After 2 hours	3.90 ± 2.70	4.83 ± 2.82	0.288
After 6 hours	4.5 ± 3.23	5.03 ± 2.90	0.380
After 8 hours	2.93 ± 2.27	3.73 ± 2.91	0.172

## Discussion

Diclofenac sodium is a nonselective cyclooxygenase inhibitor. On the other hand, ketoprofen is a relatively newer medication that has been clinically tested and found to be safe as it has no impact on platelet functions. While NSAIDs have been shown to be beneficial, paracetamol alone is ineffective for treating acute postoperative pain. Previous studies have suggested that both ketoprofen and diclofenac have better effects on early postoperative pain when compared to paracetamol [15]. The administration of analgesics via the intramuscular route has been supported in the published literature [17]. After coming out of local anesthesia, there was minimal distress, which made it much easier to handle the patient in the early postoperative period [18]. Additionally, there was a tendency for ketoprofen to provide better pain relief during the first week following surgery. Using medications with a lengthy half-life for postoperative analgesia provides similar advantages [19].

A study conducted by Niemi et al. highlighted that diclofenac 1 mg/kg given 30 minutes prior and four hours after the surgery gives better pain control when compared to ketoprofen 1.35 mg/kg [20]. Oxycodone was utilized as a rescue drug in this study under general anesthesia, and it was shown that the diclofenac group required less oxycodone than the ketoprofen and saline groups did.

Seymour et al. [21] documented that ketoprofen 25 mg is more effective at managing postoperative pain following third molar surgery than acetaminophen 500 and 1,000 mg. Buffered ketoprofen 12.5 mg showed a significant reduction in pain and had an earlier onset of pain relief when compared to ibuprofen 200 mg [22]. In addition, Sunshine et al. reported that compared to ibuprofen 200 mg, ketoprofen 12.5 and 25 mg considerably reduced pain and had a quicker onset and shorter half-life [23]. The findings of this research are therapeutically significant because acetaminophen and ibuprofen are the two most commonly used in alleviating postoperative pain after impacted tooth removal. 

However, Olmedo et al. demonstrated that after third molar surgery, ketoprofen 50 mg and placebo produced superior analgesic efficacy than ketorolac 10 mg and 20 mg [24]. According to studies, patients who took ketorolac experienced better pain alleviation than those who took ketoprofen or a placebo and they also needed fewer rescue analgesics. However, after having a third molar extracted, diclofenac was found to provide pain alleviation comparable to that of paracetamol, ibuprofen, and ketorolac [25-27]. 

Ketoprofen additionally blocks the arachidonic acid cascade's lipoxygenase pathway, which inhibits the production of leukotrienes [28]. It prevents the release of lysosomal enzymes by stabilizing the lysosomal membranes against osmotic damage and causes tissue degradation in inflammatory reactions. It is also a potent inhibitor of bradykinin, a key chemical mediator of pain and inflammation. Sufficient research has demonstrated that ketoprofen inhibits central prostaglandin formation, which also inhibits the brain enzymes COX and nitric oxide synthase [29]. Due to ketoprofen's high level of lipid solubility, it easily crosses the blood-brain barrier within 15 minutes. Ketoprofen has recently been shown to interact with the serotonergic system in addition to inhibiting prostaglandin formation in the central nervous system. All of these processes for ketoprofen could account for why it is more effective than other NSAIDs.

Limitations of the study

The small sample size and absence of a placebo group were the limitations of this study. Placebo analgesia has been proven to play an important role in the pain alleviation experienced by patients. However, this fact does not invalidate the factors that predicted analgesia of the drugs used in our study or the study results. The future scope of the study would be to evaluate results on the larger sample size and assess the effectiveness of these drugs as a pre-emptive analgesic. 

## Conclusions

In recent times, day-care surgery is currently being promoted for medical, social, and financial reasons. Therefore, the importance of NSAIDs in postoperative pain management is of utmost importance. It can be concluded from the study that the postoperative pain reduction was comparable between the two groups - ketoprofen and diclofenac sodium. However, ketoprofen group patients have lesser pain perception compared to the diclofenac sodium group patients. In future, multi-centric studies with larger sample size are required to validate this finding as well as the timings of administration, either preoperatively or postoperatively.
